# Ethical and deontological aspects of pediatric biobanks: the situation in Italy

**DOI:** 10.1007/s10561-020-09833-4

**Published:** 2020-04-20

**Authors:** Nunzia Cannovo, Rosa Guarino, Piergiorgio Fedeli

**Affiliations:** 1 Ethics Committee of University of Naples ‘Federico II’, Via Sergio Pansini 5, Naples, Italy; 2 University of Naples ‘Federico II’, Naples, Italy; 3 Law School, University of Camerino, Via A. D’Accorso 16, Camerino, MC Italy

**Keywords:** Pediatric biobanks, Italy, Genetics research, Regulation

## Abstract

While pediatric biobanks are a precious resource for scientific research to improve our understanding of genetic pathologies, the value of these studies should be considered together with the value of the privacy rights of pediatric donors, as they are particularly vulnerable and in many cases unable to discern the meaning of the donation of biological material and the related implications of the research. Thus this work calls for reflection on the numerous ethical and legal issues involved in the development and regulation of these biobanks. In particular, it explores what form of consent best balances the intangible rights of the minor, on the one hand, and the development of technological progress and scientific research, on the other, and examines the implications of the collection of biological material of minors in biobanks. It focuses on solutions to bridge the gaps in current Italian legislation, especially in light of the current lack of attention to the interests of fragile subjects. In addition, this work presents an overview of the pediatric biobanks in Italy.

## Introduction

Biobanks are an important resource for research, and in particular, pediatric ones can provide useful information for understanding the interactions between genetics and the onset of certain pathologies, enabling the development of studies about multifactorial pathologies and the achievement of personalized medicine (Brothers 2011).

In particular, biological material stored in biobanks, in relation to the progress in research on the identification of genes and diseases, has made it possible to ascertain the causes of hereditary pathologies in family units (for example mitochondrial disorders and resistant epileptic encephalopathies), to learn about specific mutations, to prepare diagnostic tests to identify those with the disease and those who are bearers (Wright et al. 2018), to evaluate the risk of procreating, and to make possible prenatal diagnosis (Dagna Bricarelli 2011).

A prime example of the value of translational genomics is the NIH-funded Clinical Sequencing Exploratory Research (CSER) Consortium, eMERGE (Electronic Medical Records & Genomics) Network, and PGRN (Pharmacogenomics Research Network) projects, that aim to research and develop best practices for translating genomics into clinical application (Wolf et al. 2015).

Pediatric biobanks are a relatively new invention (Secretary's Advisory Committee 2010; Provincial Health Services Authority 2010; Nørgaard-Pedersen and Hougaard 2007; Gurwitz et al. 2009), given the degree of protection always extended to research on minors. It should be noted that research on pediatric biological material does not always directly benefit the minor donor, and thus it is not possible to use the same precautions dictated for recruiting minors for clinical trials (EU Regulation n. 536/2014).

In the case of a research study that has no direct benefit to minors, in order to request the use of their biological samples or data, it is indispensable to explain the rationale and demonstrate that the research cannot be conducted with adult subjects. In contrast, for studies on pediatric genetic material, these precautions are reductive and not appropriate to the particular characteristics of the research.

In the bioethics literature of recent years, the participation of minors in biobanks has been the subject of intense debate on numerous bioethical questions and dilemmas, given that minors are particularly vulnerable subjects (Toccacelli et al. 2014).

The present work deals specifically with the Italian reality, focusing on the ethical and deontological issues related to biological material obtained from neonatal screening or residual parts of samples (Presidential decree 1999) taken for pediatric diagnostic tests or treatments, and later destined for research (Knoppers et al. 2012). This work also provides an overview of the situation of biobanks in Italy. In general, inquiry related to the ethics of tissue sample storage for genetic research has focused on the need for a valid consent form (Knoppers et al. 2012) that provides an exhaustive and understandable explanation of the research goals, strategies for protecting privacy, methods for communicating individual results (for example, confirmation of the illness identified), information about the commercialization of the results, and finally, the length of time the sample will be kept and the methods for its destruction (Bin et al. 2018a, b).

These already complex issues are further complicated in the case of minors because research using pediatric biobanks poses additional ethical problems (Samuël et al., 2012). In fact, minors have limited capacity to understand the meaning and implications of the research, as well as to express informed consent, as these capacities are acquired only gradually (Conti et al. 2018).

The acquisition of biological tissues from minors for storage in biobanks and use in research has very important positive consequences for science and health, but these goals cannot take precedence over the rights and interests of individual subjects involved in the research.

The present work does not dwell on the donation of organs, tissues and cells from living or deceased subjects for transplants, because this is regulated by a specific and very strict set of regulations regarding the treatment goals for which the biobank was established (Law 91/1999), which fall outside the issues the authors intend to address in this work.

In the interests of completeness, though, we will look briefly at Law 91/1999, which stipulates that in the case of minors, the will to donate organs is manifested by the parents who have authority as such; should the two parents disagree about donating their child’s organs, it is not possible to declare the willingness to donate. In addition, the law does not allow manifestation of willingness to donate organs from the unborn or from minors entrusted to or hospitalized in public or private institutions of assistance.

This article does not discuss the establishment of biobanks for medically assisted procreation (Law 4/2004) for a couple, or in the case of surrogate motherhood (the latter is not allowed in Italy) (Casella et al. 2018), as these issues are beyond its purview.

## Types of biological samples to use, and methods for obtaining them: juridical aspects

In general, according to Brothers, biobanks (Brothers 2011) can be organized into the following four groups: biobanks that actively collect disease-specific samples and information; biobanks that actively collect samples and information from all persons without regard to disease status; biobanks that passively collect disease-specific samples and information; and biobanks that passively collect samples and information from all persons without regard to disease status.

Pediatric biological samples collected for genetic research come from many diagnostic and treatment practices, as is also the case for adults (blood tests, residual biological material after drying, etc.) (Cambon-Thomsen 2004).

Recent Italian legislation (Law 3/2018) granted the opportunity to use biological or clinical material remaining from previous diagnostic or treatment activities, or kept for any other purposes, on the condition of obtaining the patient’s informed consent beforehand.

This law further states that structures where clinical experimentation is carried out should facilitate the use of residual biological or clinical material for clinical research purposes.

In subsequent legislation, the Higher Institute of Healthcare (Legislative decree n. 52/2019) was assigned the responsibility for defining homogeneous criteria for the use of biological samples, taking into consideration the methods of access to and acquisition of patient consent for the subsequent use of the sample taken. It is to work with the support of the Biobanking and BioMolecular Resources Research Infrastructure (BBMRI), and must consider the opinions of the Center of National Coordination of Local Ethics Committees and of the Guarantor for the Protection of Personal Data.

In this context, special concerns emerge regarding samples obtained through obligatory neonatal screening tests to promote the diagnosis and treatment of some genetic diseases (Law 104/1992; Presidential decree 1999), when current law requires the conservation of residual material from the obligatory testing (Law 104/1992; Presidential decree 1999) In fact, issues of professional liability may come into play in connection with this activity (Bin et al. 2018b) These samples, which normally would be eliminated according to methods stipulated by law, can be collected in a biorepository (Decree of the President of the Republic n. 254/2003).

There is an important distinction between samples that are initially used for research with parental permission and those newborn screening samples that are typically collected in most states without parental permission; such screening programs will face similar issues with the continued use of those samples that may shed light on the underlying issues for pediatric biobanks (Goldenberg et al. 2009).

In sum, the distinction between samples used for research with parental permission and those that are obtained routinely from all newborns is the purpose for which they were collected.

Italy has no ad hoc regulations on pediatric biobanks (Fedeli et al. 2019a), as the matter is covered by privacy laws. However, current practice is heterogeneous and irregular, and, given the importance and delicacy of the issue, there is need for legislation that provides homogeneity and certainty (Salvaterra 2012).

The debate became even livelier with the introduction of legislation obligating healthcare structures that carry out clinical experimentation to make available for clinical research the biological or clinical material left over from previous diagnostic or treatment activities, or kept for any reason (Legislative Decree n.52/2019).

### Prohibition of financial gain from samples

The Oviedo Convention (art. 21) (Piciocchi 2001) states, “The human body and its parts shall not, as such, give rise to financial gain” (Ferrando 2002; Zatti 1994; Gambaro 1990; Messinetti 2001; Venuti 2001; Galasso 2001; D’Arrigo 2005; Piria 1990), a principle also articulated in the opinion of the National Committee for Bioethics (2014). Similarly, the European Union Charter of Fundamental Rights, article 3, in the sphere of medicine and biology, prohibits the use of the human body and its parts as such for financial gain (Carta dei diritti fondamentali dell’Unione Europea n. 2000/C 364/01). This prohibition certainly covers pediatric biobanks as well.

In this context, as noted by the National Committee for Bioethics (2009), research biobanks play a significant role as entities able to balance the interests and rights of those involved. In addition, they must be a “new instrument of social solidarity” based not only on voluntary sharing of samples and information, but also on respect for the intangible values of the individual. Efforts should be made to make parents aware of the importance of consenting to the use of biological samples as a gesture of solidarity.

### The use of biological samples

The protection of the rights of the person, especially that of personal privacy, is one of the most delicate aspects in the management of a biobank, and even more so when it is a pediatric biobank.

Treatment of genetic data and use of biological samples is allowed for purposes of scientific and statistical research when it is closely related to the goals pursued, which must entail a direct benefit for the donors of biological samples (Authorization n. 8/2016).

The minor should benefit from the knowledge and results obtained through the research (WMA 2013). However, when certain conditions hold, the data and biological samples of minors can be used for scientific research that does not have direct benefit for the donors, specifically, when

- The research seeks to improve the health of others in the same age group or who suffer from the same pathology, or who are in the same conditions, and the research program has received the well-considered approval of the local ethics committee;
- Research with analogous goals cannot be conducted through treatment of data from people who can provide consent;
- Consent for data use is given by those with legal authority, that is, a spouse, a family member, or, in their absence, the director of the structure where the person is staying;
- The research does not present significant risks for the fundamental dignity, rights and freedoms of the persons involved.

In these cases, there remains the need to obtain the opinion of the minor, whenever possible.

## Informed consent

In Italian law (Fedeli et al. 2019b), informed consent is the expression of the freedom of self-determination of the person in relation to acts concerning his or her body (Di Lorenzo 2018), grounded in the Constitution of the Republic (articles 2, 13, and 32). In addition, the essential need to protect minors, given their particular vulnerability, must be considered. The law of December 22, 2017, n. 219, “Regulations on the matter of informed consent and provisions prior to treatment” (Law 219/2017) established the limitations to and methods for obtaining consent, but provided no specific reference to issues involved in obtaining the consent of a minor. Thus consent for minors is granted by parents or a legal representative (Legislative Decree n.154/2013), who must declare their consent (or better, permission) (Rossi et al. 2003) at the moment of granting and/or donating the minor’s biological material to the biobank. The theme of the informed consent of minors is particularly problematic, given that until they acquire the capacity for discernment, minors are not able to give valid consent regarding the present or future destiny of their own biological material.

In the literature, there is general agreement that minors must be involved in all three steps of collection and inclusion of the sample, storage of the sample, and usage of the sample, in relation to their degree of maturity (Giesbertz et al. 2016).

Similarly, the Guarantor for the Treatment of Personal Data has established that when the minor’s age and degree of maturity allow it, his or her opinion should be taken into consideration, with primary attention to “the best interests of the minor” (Authorization n. 8/2016).

Given the evident importance of the information to be provided to the minor and parents or legal representatives, it is essential that this it be as complete as possible, detailing the goals of the scientific project in which the sample is to be used, how long it will last, where it will take place and the methods that will be used.

In addition, it should be specified that in relation to the provisions of Italian law, the choice to donate the biological material of one’s child certainly constitutes an act of extraordinary administration, which requires the explicit consent of both parents, in accordance with article 320 of the Civil Code. The directives of the Guarantor, however, stipulate that consent of both parents is required only for genetic testing for individual variability (Authoritation n. 8/2016).

The process of defining a form of consent that balances protection of the intangible rights of the minor and the development of scientific research is particularly delicate. As the directly involved subject does not provide the consent, and as the effects of donating the minor’s biological samples may not be neutral, the National Committee for Bioethics (2014) asserts that the samples must not be irreversibly anonymized, and the authorization of the parents or legal representative should not be “broad,” but rather, should be given for a specific research project. Alternatively, they can give “partially restricted consent,” directly correlated with the research project, after having been provided complete, detailed information, so the donors can evaluate the goals of the scientific project in which the sample is to be used, how long it will last, where it will take place and the methods that will be used. Thus the parents retain “control” over the use of the biological material of their child, and can ask for further information and, should they revoke their consent, can request the destruction of the biological sample and associated biographical and clinical data.

In order to avoid stigmatization of or discrimination against the minor, it is indispensable that the genetic consultation provide detailed information on the modalities of treatment of the genetic data, indicating the confidentiality of the results, the goals for which it is used, and the methods for accessing the information.

Should there be a risk that the minor can be identified, the parents, as the legal guardians, must be promptly informed (Palazzani 2017). The rationale for this provision is that a minor is a weak and vulnerable subject who must be protected.

The National Committee for Bioethics (2014) stresses that the biobank has the responsibility to explicitly tell the parents to inform their child about the donation and to maintain contact with the biobank so that the minor may become a party to the consent. The parent’s role is particularly delicate in deciding what, when and how to tell the child about the donation.

In addition, the minor must be informed at the moment of providing the samples (Yu et al. 2013), taking into consideration the degree of maturity reached (Brothers and Goldenberg 2016) and the capacity to understand, given that the minor’s possible refusal outweighs the permission given by the parent or legal representative. The correct management and use of material granted to a pediatric biorepository must be based on the essential rule that the importance of parental permission should gradually fade while that of the minor comes to the fore, through a process to provide a voice to the adolescent’s claims as she or he progresses toward maturity. When donors reach the age of legal authority, they must promptly be given the opportunity to indicate their consent (Ross 2006) or, if the research is already underway, to renew, modify or revoke the consent to the use of their samples and data in the biobank (Authoritation n. 8/2016).

The possibility that the parents’ consent to the use of their child’s biological sample can remain valid even when that child reaches adulthood presents a particularly complex problem (Goldenberg et al. 2009). The debate certainly contemplates the possibility of continuing the scientific research or instead interrupting it in order to request permission once again, this time from minors who have come of age (Brothers 2011), in order not to undermine their self-determination or violate their right to privacy.

While the Guarantor calls for an effort to obtain new consent, it has also contemplated the practical difficulties researchers could face in re-contacting participants (Authoritation n. 8/2016), in particular when particular or exceptional factors make it impossible to inform the donors, or demand a disproportionate effort, or risk gravely compromising or even rendering impossible the achievement of the research goals.

It follows that those responsible for pediatric biobanks (guarantors or curators) must develop procedures and appropriate instruments for contacting donors when they come of age, so the latter can obtain adequate information, gain access to the samples and data, and if they so desire, rescind the consent, have the samples destroyed and/or have the information eliminated (National Bioethics Committee 2014).

Ethics committees play a central role when proposals are submitted that entail sample collection methods that can harm the present or future interests of minors, or that involve non-conventional studies (Brothers and Goldenberg 2016). The Guarantor (Authoritation n. 8/2016) calls for Ethics Committees to take an active role in situations in which it has not been possible to inform the donors, even when every reasonable effort has been made to contact them.

## Communication and sharing of data

The unique aspect of genetic research on biological samples is founded on the possibility of obtaining biological information but also on the correlation with eventual clinical conditions that may develop, thus it appears fundamental to be able to interrelate these data.

Therefore, biobanks need regulations that guarantee specific protection of privacy.

Italian law forbirds the sharing of genetic data. Research results cannot be shared, except in aggregated form, that is, in such a way that the donors cannot be identified, not even through indirect identification data, including the sphere of publications (Authoritation n. 8/2016).

The results of diagnostic tests involving biological samples cannot be communicated to employers (Cannovo et al. 2010) or insurance companies (Bin et al. 2018d), but used for diagnostic purposes or those of scientific research.

Genetic data (Authoritation n. 8/2016) and biological samples collected for purposes of scientific and statistical research can be communicated or transferred to research entities or institutes, associations and other government and private organisms for research, exclusively in the context of joint projects. However, information without identification data can be made available to third parties that are not participants in joint projects, for scientific goals directly linked to those for which they were originally collected and clearly defined in writing in the request for data and/or samples. In this case, the subject making the request commits to not using the data and/or samples for purposes different from those indicated in the request, and to not communicating or transferring them to others.

## Pediatric biobanks in Italy

In Italy, most of the biobanks and centers of biological resources are found at structures or institutions that are part of or connected to the national healthcare service, for example, hospitals and special research institutes for hospitalization and treatment (IRCCS). Since 2009 they have operated subject to regional regulations (Conferenza delle regioni e delle province autonome 2009).

Generally, they are partners in national, European, and international networks.

In a joint effort, the Italian Ministry for Universities and Research and the Health Ministry established the national node of the European Biobanking and BioMolecular Resources Research Infrastructure (www.bbmri-eric.eu). The national node includes 80 Biobanks, Centers of Biological Resources and Collections in various regions of Italy, as well as 4 Common Services (a Quality Management CS, an Information Technology CS, an Ethical, Legal and Social Implications project (ELSI) CS for ethical, legal and social issues, and a CS for training), which collaborate with research institutes for hospitalization and treatment (IRCCS) (https://www.salute.gov.it/portale/temi/p2_6.jsp?id=794&area=Ricerca%20sanitaria&menu=ss), hospitals, the National Center for Research (CNR), and its institutes and associations of patients.

Table 1 lists the biobanks with samples prevalently from the pediatric population.

**Table 1:** Biobanks with pediatric population

Biobank name	Location	Types of pathologies studied
Banca Biologica Oncologica Pediatrica [Pediatric Oncological Biobank]	Dip. Salute della Donna e del Bambino [Department of Health of Women and Children]	Lymphomas, solid tumors and brain tumors
Cell line and DNA Biobank from patients affected by Genetic Diseases	Istituto Giannina Gaslini [Giannina Gaslini Institute]	Rare genetic diseases
Cell lines and DNA bank of Paediatric Movement Disorders and Neurodegenerative Diseases	Fondazione IRCCS Istituto Neurologico Carlo Besta [Carlo Besta Neurological Institute, IRCCS Foundation]	Movement Disorders and Neurodegenerative Diseases
Biobanca Integrata Tessuto-Genomica [Integrated Tissue and Genomic Biobank]	Istituto Giannina Gaslini [Giannina Gaslini Institute]	Miscellanea
Banca biologica pediatrica [Pediatric Biological Bank]	Clinica di Oncoematologia Pediatrica di Padova [Pediatric Oncohematology Clinic, Padova]	Miscellanea
Biobanca Di Ricerca OPBG [OPBG Research Biobank]	IRCCS Ospedale Pediatrico Bambino Gesù [Baby Jesus Pediatric Hospital IRCCS]	Miscellanea
Biobanca dei tumori solidi in età pediatrica [Biobank of solid tumors in pediatric age]	Ospedale Pausillipon di Napoli [Pausillipon Hospital of Naples]	Neuroblastomas and pediatric tumors
Biobanca ad elevati standard tecnologici per indagini biomolecolari nell’ambito della ricerca medico-scientifica [High technological standard biobank for biomolecular studies in medical and scientific research]	Dipartimento di scienze clinico-chirurgiche, diagnostiche e pediatriche di Pavia [Department of Clinical, Surgical, Diagnostic and Pediatric Sciences, Pavia]	Miscellanea
Telethon Network of Genetic Biobanks	ISTITUTO GIANNINA GASLINI [Giannina Gaslini Institute]	Genetic diseases

The biobanks are located at pediatric healthcare structures and the sphere of interest is prevalently congenital diseases and oncohematology pathologies.

In fact, congenital diseases are the third cause of hospitalization of minors in Italy (Corsello et al. 2013), where between 2003 and 2008, every year on average there were 164 diagnoses of malignant tumor for every million children (0–14 years old) and 269 cases for every million adolescents (ages 15–19) (https://www.airc.it/pediatrici).

## Conclusions

The opportunity to conduct research on pediatric biological samples has had a notably positive impact in improving knowledge about genetic diseases, with benefits to both diagnosis and treatment.

It has been possible to identify rare diseases, pediatric-specific disease markers, and hereditary conditions, and thus to prepare diagnostic tests to identify those affected and bearers, and to develop specific treatments.

As minor donors come of age, they should be contacted through new computer technologies to obtain their informed consent to the donation of biological material that their parents or legal representatives had authorized.

Biobanks struggle with a range of practical and ethical issues related to this question.

We believe that it is necessary to balance the rights of minors, a vulnerable population, with the needs of scientific research in the age of genomic medicine.

Italy hosts a small number of pediatric biobanks, but the number should increase with the introduction of new legislation, especially Legislative Decree 52/2019, which allows the use of biological material left over from diagnostic and treatment activities.

It is to be augured that ad hoc regulations that balance the rights of those involved will be forthcoming, as current legislation on sensitive data is not sufficiently specialized in this regard.

We are also convinced that the modifications to the Authorizations issued by the Guarantor n. 8/16 and 9/16, after the issuance of the EU Regulation 2016/679, will afford greater flexibility in the use of genetic data and biological samples from populations of minors, thus allowing this sector of the population, often excluded from clinical trials, to benefit from the advantages offered by pharmacogenetics and pharmacogenomics.
